# Identification of a Clinical Cutoff Value for Multiplex KRAS^G12/G13^ Mutation Detection in Colorectal Adenocarcinoma Patients Using Digital Droplet PCR, and Comparison with Sanger Sequencing and PNA Clamping Assay

**DOI:** 10.3390/jcm9072283

**Published:** 2020-07-18

**Authors:** Kyung Ha Lee, Tae Hee Lee, Min Kyung Choi, In Sun Kwon, Go Eun Bae, Min-Kyung Yeo

**Affiliations:** 1 Department of Surgery, Chungnam National University Hospital, Daejeon 282, Korea; lllllkh@cnuh.co.kr; 2 The Biobank of Chungnam National University Hospital, Daejeon 282, Korea; biotech89@cnuh.co.kr; 3 Department of Pathology, Chungnam National University School of Medicine, Daejeon 266, Korea; mk6214@hanmail.net (M.K.C.); goeunbae1@gmail.com (G.E.B.); 4 Clinical Trials Center of Chungnam National University Hospital, Daejeon 282, Korea; kiss95@cnuh.co.kr

**Keywords:** colorectal cancer, ddPCR, cutoff, ROC, PNA-clamping assay, Sanger sequencing

## Abstract

KRAS (Kirsten rat sarcoma 2 viral oncogene homolog) is a major predictive marker for anti-epidermal growth factor receptor treatment, and determination of KRAS mutational status is crucial for successful management of colorectal adenocarcinoma. More standardized and accurate methods for testing KRAS mutation, which is vital for therapeutic decision-making, are required. Digital droplet polymerase chain reaction (ddPCR) is an advanced digital PCR technology developed to provide absolute quantitation of target DNA. In this study, we validated the clinical performance of ddPCR in determination of KRAS mutational status, and compared ddPCR results with those obtained by Sanger sequencing and peptide nucleic acid-clamping. Of 81 colorectal adenocarcinoma tissue samples, three repeated sets of KRAS^G12/G13^ mutation were measured by ddPCR, yielding high consistency (ICC = 0.956). Receiver operating characteristic (ROC) curves were constructed to determine KRAS^G12/G13^ mutational status based on mutant allele frequency generated by ddPCR. Using the best threshold cutoff (mutant allele frequency of 7.9%), ddPCR had superior diagnostic sensitivity (100%) and specificity (100%) relative to the two other techniques. Thus, ddPCR is effective for detecting the KRAS^G12/G13^ mutation in colorectal adenocarcinoma tissue samples. By allowing definition of the optimal cutoff, ddPCR represents a potentially useful diagnostic tool that could improve diagnostic sensitivity and specificity.

## 1. Introduction

Colorectal cancer (CRC) is one of the most common cancers in the United States [1]. Approximately 130,000 CRC patients are newly diagnosed each year, and nearly 30% of these patients develop distant metastasis. In 2017, a total of 50,260 CRC patients died in the United States [2,3]. Following the emergence of monoclonal antibodies against epidermal growth factor receptor (EGFR) and vascular endothelial growth factor (VEGF), cytotoxic chemotherapy plus targeted monoclonal antibody has been recommended as the standard treatment for metastatic CRC around the world [4]. Anti-EGFR monoclonal antibody is recommended for RAS–wild or left colon cancers, and VEGF antibody is recommended for RAS-mutant or right colon cancers. Accordingly, the mutational status of EGFR downstream signaling effectors, especially KRAS, must be screened to determine the ideal course of targeted therapy.

KRAS is a major predictive marker for anti-EGFR treatment, and determination of KRAS mutational status is crucial for successful management of CRC patients. Mutation of KRAS is detected in 35–45% of CRC patients, and most of these mutations affect codons 12 and 13 [5]. Multiple KRAS detection methods have been investigated, including direct sequencing, pyrosequencing, polymerase chain reaction (PCR) with peptide nucleic acid (PNA)-mediated clamping, and next-generation sequencing (NGS) [6,7,8]. To detect KRAS mutation in a more effective manner, effort has been devoted to decreasing the amount of tumor material required, and optimizing the sensitivity and specificity of testing.

Digital droplet PCR (ddPCR) is a recently developed sequencing method capable of sensitively detecting target DNA in varying backgrounds of wild-type DNA in a small amount of material [9]. By partitioning DNA into a large number of droplets, ddPCR can provide absolute quantification and detect target DNA in a much higher background of non-target (usually wild-type) DNA. Due to its technological advantages, ddPCR has been adopted for testing of KRAS mutation in patient samples [4]. However, the clinical performance of ddPCR-based KRAS mutation detection in CRC has not been carefully evaluated.

In this study, we sought to validate a ddPCR platform for detection of KRAS^G12/G13^ mutation (KRAS codon 12 and codon 13) using CRC patient tissue samples. KRAS^G12/G13^ mutant allele frequency (MAF) generated by ddPCR was measured repeatedly to assess the consistency of ddPCR. Receiver operating characteristic (ROC) curves were constructed based on the MAF to determine KRAS^G12/G13^ mutational status with the optimal cutoff value. The diagnostic performance of ddPCR was compared with gold standard methods for KRAS^G12/G13^ mutational analysis: Sanger sequencing and PNA-clamping assay (PCR with PNA-mediated clamping).

## 2. Materials and Methods

### 2.1. Sample Selection and DNA Isolation

This study was performed using 81 formalin-fixed paraffin-embedded (FFPE) surgically resected colorectal adenocarcinoma (CRAC) tissue samples collected at a single institutional center (Chungnam National University Hospital, Daejeon, South Korea) from January 2014 to December 2017. Tissue samples included primary and metastatic (liver, lung, and ovary) tissues from CRAC patients. Hematoxylin/eosin-stained slides of selected cases were pathologically reviewed by two pathologists (M-KY and GEB), and the most representative areas were selected. Twenty non-neoplastic colon FFPE tissue samples (10 samples acquired from tissues located more than 5 cm apart from the CRAC and 10 samples from surgically resected colon due to inflammation) were included for negative control. Sixteen serums from healthy persons were also included negative control.

Twenty-micron thick sections of FFPE samples were prepared and deparaffinized in xylene. DNA was isolated using the QIAamp DNA FFPE Tissue Kit and QIAamp Circulating Nucleic Acid kit (QIAGEN Korea, Seoul, South Korea). All extracted DNA was diluted to 10 ng/μL. Extracted DNAs were evaluated for KRAS^G12/G13^ mutation using ddPCR, Sanger sequencing, and PNA-clamping PCR. All bio-specimens and data used for this study were provided by the Biobank of Chungnam National University Hospital, a member of the Korea Biobank Network. The study was approved by Chungnam National University Hospital institutional review board (IRB file no. 2018-10-012-001). The study was retrospective, and a waiver of consent was approved by the Institutional Review Board.

### 2.2. Droplet Digital Polymerase Chain Reaction (ddPCR)

**Figure 1:**
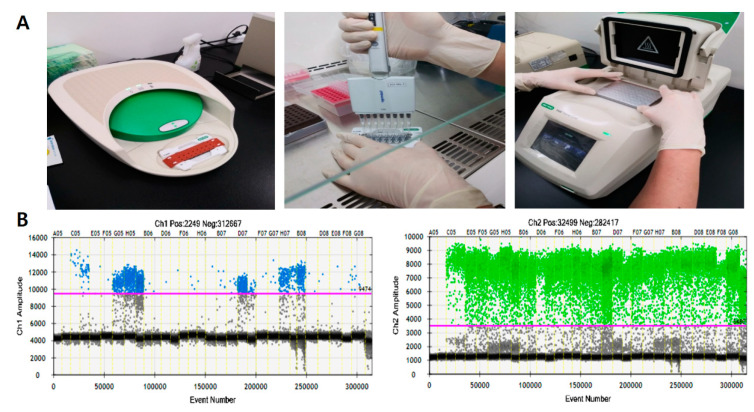
Droplet digital polymerase chain reaction (ddPCR) workflow (**A**) and representative results of ddPCR for detection of KRAS^G12/13^. Channel 1: fluorescence measurement of mutant probe labeled with 6-fluorescein amidite (FAM). ((**B**) left) Channel 2; wild-type probe labeled with hexachlorofluorescein (HEX) ((**B**) right).

Extracted DNA from CRAC tissue samples was tested with ddPCR (QX200; Bio-Rad, Hercules, CA, USA) using the ddPCR Bio-Rad KRAS^G12/G13^ multiplex kit (#1863506) for screening of codons 12/13 (Figure 1A). Reaction mixtures (final volume, 20 µL) consisted of extracted DNA (1 μL), 2× SuperMix for probe (10 μL), KRAS screening probe (1 μL), and distilled water (8 μL). The mixture was loaded into a disposable droplet generator cartridge (Bio-Rad), and 70 μL droplet generation oil for primer (Bio-Rad) was loaded into each of the eight oil wells. The cartridge was then placed inside the QX200 droplet generator (Bio-Rad), which partitioned each tissue sample into ~22,000 droplets per tissue sample. When droplet generation was completed, the droplets were transferred to a 96-well PCR plate. The plate was heat-sealed with foil and placed in a conventional thermal cycler (T100, Bio-Rad) using the following reaction conditions: 95 °C for 10 min (1 cycle); 94 °C for 30 s and 55 °C for 1 min (40 cycles); 98 °C for 10 min (1 cycle); and 4 °C hold. Cycled droplets were read individually on a QX200 droplet-reader (Bio-Rad). Samples were transferred to the QX200 for fluorescence measurement of mutant probe labeled with 6-fluorescein amidite (FAM) and wild-type probe labeled with hexachlorofluorescein (HEX). DNA from SW480 cell line (KRAS G12V mutation) served as a positive control; DNA from the leukocytes of the heathy persons, HEK cell line, and distilled water were used as negative control, respectively.

The QuantaSoft software (version 1.7; Bio-Rad) classifies droplets by first determining a fluorescence threshold (Figure 1B). Some droplets were in the intermediate “rain” (gray droplets), which had fluorescence ranging between those of explicit positive and negative droplets. The dashed horizontal line in Figure 1B indicates a fluorescence value greater than the set threshold; these were considered positive [10]. After analyzing the number of positive and negative fluorescence signals in droplets, MAF was calculated as the percentage of mutant droplets relative to the total (mutant + wild-type).

### 2.3. Sanger Sequencing

Extracted DNA (20 ng) from CRAC tissue samples were sent for Sanger sequencing (Macrogen, Seoul, Korea).

For mutation analyses in codons 12 and 13 of the KRAS gene, primer sequences for exon 2, 5′-GTAAAACGACGGCCAGTGTGTGACATGTTCTAATATAGTCA-3′(forward) and 5′-GCGGATAA CAATTTCACACAGGGAATGGTCCTGCACCAGTAA-3′ (reverse) and for exon 3, 5′-TAATA CGACTCACTATAGGGGTGCTTAGTGGCCATTTGTC-3′ (forward) and 5′-GCTAGTTATTGC TCAGCGGTATGCATGGCATTAGCAAAG -3′ (reverse) were utilized for the PCR reaction.

PCR amplification conditions were as follows: 95 °C 5 min; 95 °C 30 s, 60 °C 30 s, 72 °C 1 min for 35 cycles; 72 °C 7 min. PCR products were purified using Millipore plate MSNU030 (Millipore SAS, Molsheim, France). The purified PCR products were then Sanger-sequenced with the BigDye terminator v3.1 sequencing kit and a 3730xl automated sequencer (Applied Biosystems, Foster City, CA). Nucleotide sequence data were analyzed with Variant reporter computer software version 1.1 (Applied Biosystems, Foster City, CA, USA).

### 2.4. Peptide Nucleic Acid (PNA)-Clamping Assay (PCR with PNA-Mediated Clamping)

Extracted DNA (7 μL) from CRAC tissue samples was tested by PNA-clamping assay using the PNA clamp KRAS mutation detection kit (version 4; Panagene, Daejeon, South Korea) for screening of codons 12/13/59/61/117/146. Reaction mixtures contained 7 µL DNA template, 3 μL of each PNA mix, and 10 μL of 2× premix, and amplification was performed in a CFX96 real-time PCR instrument (Bio-Rad) with the following thermal program: pre-incubation at 94 °C for 5 min, followed by 40 cycles of amplification at 94 °C for 30 s (s), 70 °C for 30 s, 63 °C for 30 s, and 72 °C for 30 s.

The efficiency of PNA-mediated PCR clamping was determined by measuring the threshold cycle (Ct) value. The Ct values for the control and mutation assays were obtained by observing the SYBR Green amplification plots. The delta Ct (ΔCt) value was calculated ([Control Ct] − [Sample Ct] = ΔCt) and the cutoff ΔCt was defined as 2 for the all mutations.

### 2.5. Statistical Analysis

To develop ROC curves for KRAS^G12/G13^ detection by the ddPCR platform, cases in which mutations were detected by Sanger sequencing were considered as positive references. KRAS^G12/G13^ mutation detection by ddPCR was performed three times (first, second, and third), and the mean MAF of KRAS^G12/G13^ was used to develop ROC curve. Internal consistency of the scales was assessed by Cronbach’s alpha via the intraclass correlation coefficient and kappa coefficient.

Diagnostic value (sensitivity, specificity, positive predictive value, and negative predictive value) was calculated for the detection of KRAS^G12/G13^ mutation by ddPCR, Sanger sequencing, and PNA-clamping assay. All statistical analyses were performed using SPSS version 26.0 for Windows (SPSS Inc., Chicago, IL, USA) and MedCalc version 19.2.0 for Windows (MedCalc Software Ltd., Ostend, Belgium).

## 3. Results

### 3.1. Detection of KRAS^G12/G13^ Mutation by ddPCR, Sanger Sequencing and PNA Clamping Assay

The ddPCR platform used the QuantaSoft software to measure the numbers of positive and negative droplets in each well. A total number of generated droplets ranged from 10,461 to 30,796 per well (mean: 18,182). Samples with fewer than 10,000 generated droplets were excluded from analysis. Threshold horizontal lines were set at 9474 for channel 1 (KRAS^G12/G13^ mutant) 3480 for channel 2 (KRAS^G12/G13^ wild) (Figure 1B). MAF was calculated as the percentage of mutant droplets relative to all (mutant + wild-type) droplets.

Sixteen serums from healthy persons showed no KRAS^G12/G13^ mutant droplets. Twenty non-neoplastic colon tissue samples showed 0 to 6 KRAS^G12/G13^ mutant droplets and the MAF were 0 to 0.55% (mean: 0.06%). A total of 81 CRAC tissue samples showed KRAS^G12/G13^ mutant droplets from 0 to 1121 droplets and the MAF were 0 to 81.17% (mean: 11.13%).

Sixteen serums from healthy persons and 20 non-neoplastic colon tissue samples were all KRAS^G12/G13^ wild type by Sanger sequencing and PNA clamping assay. A total of 81 CRAC, 51 (63%) CRAC were KRAS^G12/G13^ wild type and 30 (37%) CRAC were KRAS^G12/G13^ mutant (23 cases of codon12 and 7 cases of codon 13) by Sanger sequencing. A total of 81 CRAC, 48 (59%) CRAC were KRAS^G12/G13^ wild type and 33 (41%) CRAC were KRAS^G12/G13^ mutant (27 cases of codon12 and 6 cases of codon 13) by PNA clamping assay.

### 3.2. Receiver Operating Characteristic (ROC) Curves in Determination of KRAS^G12/G13^ Mutation by ddPCR

**Figure 2:**
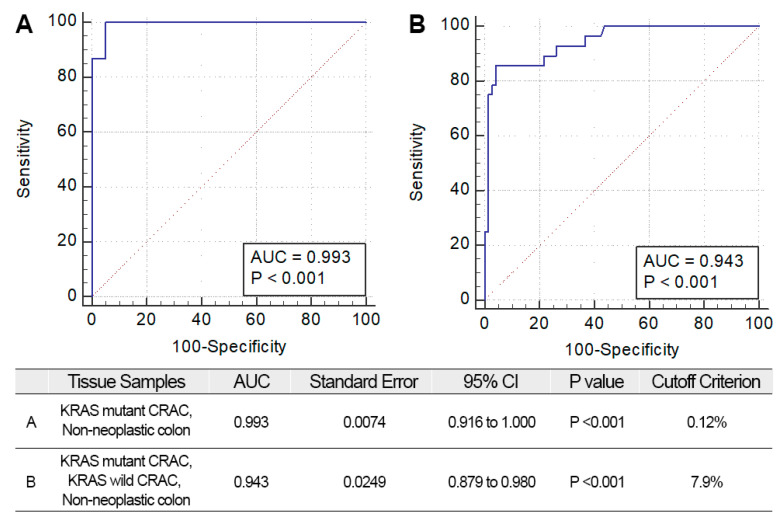
Receiver operating characteristic (ROC) curves for ddPCR. Optimal cutoff criteria and the area under curve (AUC) results are shown. (**A**) ROC curves using KRAS results from KRAS mutant CRAC and non-neoplastic colon (**B**) ROC curves using KRAS results from KRAS mutant CRAC, KRAS wild CRAC, and non-neoplastic colon.

To determine the mutational status of KRAS^G12/G13^ by ddPCR as “mutant” or “wild-type,” a cutoff value for MAF was required. To this end, we generated ROC curves to determine KRAS^G12/G13^ mutational status from MAFs generated by ddPCR (Figure 2). To develop ROC curves for KRAS^G12/G13^ detection by the ddPCR platform, cases in which mutations were detected by Sanger sequencing were considered as positive references. The negative references were used (A) non-neoplastic colon; (B) non-neoplastic colon and KRAS^G12/G13^ wild-type CRAC by Sanger sequencing (Figure 2). The AUC (area under the curve) of the A (negative reference: non-neoplastic colon) was 0.993 and optimal cutoff was 0.12% (*p* < 0.001) (Figure 2A). The AUC of the B (negative reference: non-neoplastic colon and KRAS^G12/G13^ wild CRAC by Sanger sequencing) was 0.943 and optimal cutoff was 7.9% (*p* < 0.001) (Figure 2B).

We assessed the diagnostic value of ddPCR KRAS^G12/G13^ mutation using the calculated cutoffs (Table 1). Sensitivity and specificity were 100% and 30.91% with 0.12% MAF cutoff; 84.38% and 97.96% for the ddPCR 7.9% MAF cutoff. The optimal cutoff value of the MAF was determined to be 7.9%, which yielded a maximal increase in the sensitivity and specificity.

### 3.3. Repetitive Measurement of KRAS^G12/G13^ Mutation by ddPCR

To assess the reproducibility of the ddPCR platform, we measured three sets of KRAS^G12/G13^ MAFs in CRAC tissue samples using the KRAS^G12/G13^ mutation multiplex kit. The time interval between measurements was 3 months. The means of the three MAF results were calculated. The pooled intraclass correlation (ICC) coefficient for the three sets of MAFs and the mean was 0.956 (*p* < 0.001), which is an excellent concordance rate.

With 7.9% MAF cutoff generated by the ROC curves was used to assign “mutant” or “wild-type” KRAS^G12/G13^ mutation status to the CRAC tissue samples (Table 2). In all, 74 of 81 cases (91%) yielded concordant results. The remaining 7 cases (8%) yielded discrepant results (Table 2, *).

Next, we assessed the diagnostic value of repetitive measurements of ddPCR KRAS^G12/G13^ mutation using the calculated optimal cutoffs (Table 3). Sensitivity and specificity were, respectively, 71.88% and 100% for the first measurement; 84.38% and 97.96% for the second measurement; 84.38% and 93.88% for the third measurement; and 84.38% and 97.96% for the mean.

### 3.4. Comparison of KRAS^G12/G13^ Mutation Analysis by ddPCR, Sanger Sequencing, and PNA-Clamping Assay

We validated KRAS^G12/G13^ mutation status in CRAC tissue samples by ddPCR, Sanger sequencing, and PNA-clamping assay (Table 4). Twenty-eight KRAS^G12/G13^ mutant cases were detected by ddPCR; MAF ranged from 7.9% to 81.2% (the mean mutant MAF = 30.9%). In 53 KRAS^G12/G13^ wild-type cases, MAF ranged from 0% to 7.53% (the mean of wild-type MAF = 0.67%).

Comparing two methods to detect KRAS^G12/G13^ mutation, the concordant rate of the ddPCR and Sanger sequencing was 93% (75/83); the ddPCR and PNA-clamping assay was 89% (72/81); Sanger sequencing and PNA-clamping assay was 81% (66/81). Six discordant cases (T23, T29, T38, T42, T48 and T54) were identified between ddPCR and Sanger sequencing. Except 1 case (T54), Sanger sequencing detected KRAS^G12/G13^ mutation, otherwise, ddPCR (all 3 tests were wild type) and PNA clamping assay did not detect KRAS^G12/G13^ mutation. The MAF of discordant cases were 0.17%, 0.26%, 0.39%, 0.54%, 1.78%, and 7.9%. Nine discordant cases (T15, T47, T49-T53, T59 and T61) were identified between ddPCR and PNA clamping assay. Except 2 cases (T59, T61), PNA clamping assay detected KRAS^G12/G13^ mutation, otherwise, ddPCR and Sanger sequencing did not detect KRAS^G12/G13^ mutation. The MAF of discordant cases were 0.11%, 1.04%, 2.14%, 2.42%, 4%, 5.48%, 7.53%, 18.2%, and 19.36%. T52 (MAF = 2.17%, 5.62%, and 8.65%) and T53 (MAF = 3.61%, 9.11%, and 9.87%) cases showed discordant results in 3 repetitive ddPCR results.

For comparative analysis, cases in which KRAS^G12/G13^ mutation was detected by two or more methods were defined as positive references. Twenty-eight of 81 CRAC tissue samples (35%) harbored the KRAS^G12/G13^ mutation from the results of positive references. Fifteen of 81 cases (18.5%) yielded discrepant results (Table 4, *). ddPCR generated no discordant cases relative to the positive reference. The Sanger sequencing assay yielded discordant results in 6/15 cases and PNA-clamping assay in 9/15 cases. The concordance rate (κ value) between ddPCR and the positive reference was 1.000 (*p* < 0.001); the κ value was 0.842 (*p* < 0.001) for the Sanger sequencing; 0.764 (*p* < 0.001) for PNA-clamping assay. The κ value of ddPCR with the Sanger sequencing was 0.842 (*p* < 0.001) and 0.764 (*p* < 0.001) with PNA-clamping assay.

Comparison of the diagnostic performance of ddPCR, Sanger sequencing, and PNA-clamping assay for detection of KRAS^G12/G13^ (Table 5) indicated that the sensitivity and specificity of the ddPCR test were 100% and 100%, respectively, making it superior to the other two methods (Sanger sequencing: 96.43% and 90.57% respectively; PNA-clamping assay: 92.86% and 86.79%).

## 4. Discussion

The ddPCR platform is an advanced digital PCR technology that has been used to detect and quantify target DNA or RNA in tissue or blood samples. ddPCR is a very sensitive method that can detect as little as 0.01% mutant DNA [4]. Because ddPCR provides an absolute number of fluorescent droplets, clinical applications of the ddPCR platform require delineation between positive (mutant) and negative (wild-type) KRAS^G12/G13^ mutation status, which is critical for therapeutic decision-making [11,12]. Previous studies reported various cutoffs for KRAS^G12/G13^ determined by ddPCR; Dong et al. [13] set 0.02 to 0.56% cutoffs for multiple KRAS^G12/G13^ mutation site based on detection limit on their experiments of mixing mutant KRAS^G12/G13^ DNA to wild-type DNA; Vanova et al. [14] determined an arbitrary 0.6% cutoff; Alcaide et al. [4] set a MAF cutoff of 1%, which was a threshold above background gray-zone noisy; and Laurent-Puig et al. [15] suggested a 1% threshold, which was a clinically relevant cutoff to discriminate a patient’s prognosis.

The sensitive PCR method has the possibility to lead to false positive (FP) results. An FFPE sample is very commonly used for clinical sequencing because it is easy to match tumor and normal tissue in the slides and it can be stored at room temperature. However, sequencing from DNA-extracted FFPE samples can yield errors due to fragmentation of genomic DNA and chemical processing damage to the samples [5]. The limit of detection (LOD) for detecting KRAS mutation was differently reported depending on the sample types; 0.05% for G12D and 0.01% for G12C using cancer cell lines with TaqMan MGB probes; 0.2% using FFPE CRAC samples with KRAS multiplex kit [13,16]. We detected no mutant droplets using serums samples and 0 to 0.55% MAF using non-neoplastic FFPE colon samples. With 0.12% cutoff closed to LOD, the diagnostic value of KRAS^G12/G13^ detection of ddPCR yielded a high sensitivity (100%) and low specificity (30.61%). With 7.9% cutoff generated from ROC curve validated with KRAS^G12/G13^ wild-type samples, diagnostic value of ddPCR yielded a high sensitivity (84.38%) and high specificity (97.96%). We decided that 7.9% MAF cutoff might decrease the possibility of FP results and be more appropriate for clinical application using FFPE patient samples.

Cases with wild type by ddPCR KRAS^G12/G13^ mutation have the possibility of KRAS^G12/G13^ mutant type. The sanger sequencing detected KRAS^G12/G13^ mutation in cases with low MAF (0.17%~1.78%) that 7.9% MAF cutoff can be rather high and can miss mutant cases with low MAF. We set arbitrary cutoff to increase the specificity to use ddPCR as a diagnostic assay using FFPE tissue samples. Considering the biggest merit of the ddPCR is the sensitivity, we need to set appropriate MAF cut offs depending on the sample types (blood or urine) and detection probes (TaqMan probe or multiplex kit). In a previous study, CRAC patients with below 1% MAF KRAS mutation showed more therapeutic response with anti-EGFR therapy than above 1% MAF KRAS mutation [15]. Clinical significance can also be the standard to set clinical cutoff for MAF of the KRAS mutation.

ddPCR is a fluorescent probe-based PCR assay to partition sample DNA into ~20,000 droplets that fluorescence emitted from each droplet is measured to quantitate the number of target DNA molecules [17]. The number of positive droplets is very sensitive to pipette handling during droplet generation, cartridge exchange, and minor changes in fluorescence color [18]. Taylor et al. [19] reported that ddPCR produced more consistent and reproducible data than quantitative PCR when they used samples with variable contamination. To estimate the consistency of the ddPCR platform using FFPE samples, we performed repetitive measurement of KRAS^G12/G13^. Three sets of measurements yielded seven discrepant results that 5 discordant cases raised at the first measurement and 2 cases were small-sized specimens (less than 1 cm). We guessed that an unaccustomed technique in pipette handling during droplet generation and small-sized specimens could be the reason. Low fractions of mutant DNA raised the possibility of false-positive or false-negative results. When using FFPE tissue samples, sensitivity could be limited by the amount of mutant DNA; however, in such a case, repetitive measurement could guarantee the results and increase diagnostic sensitivity. Statistically, ddPCR yielded an excellent intraclass correlation, allowing us to conclude that the ddPCR platform has the potential to be used as a sensitive and reliable method to detect KRAS^G12/G13^ mutation.

This study has some limitations. First, when developing ROC curves to calculate ddPCR cutoffs, we defined positive references based on results from Sanger sequencing. However, the mutational status assessed by Sanger sequencing did not represent the true properties of KRAS^G12/G13^ DNA in samples. However, the Sanger sequencing has been employed in diagnostic laboratories and is considered a gold standard for evaluating KRAS^G12/G13^ with high specificity. In addition, PNA-clamping is also commonly used in diagnostic laboratories for evaluating KRAS^G12/G13^ with high specificity [20]. Comparing two methods to detect KRAS^G12/G13^ mutation, the concordant rate of the ddPCR and Sanger sequencing was 93% and the ddPCR and PNA-clamping assay was 89%. Sanger sequencing detected KRAS^G12/G13^ mutation in cases with low MAF by ddPCR (mean MAF = 0.4%). PNA clamping assay detected KRAS^G12/G13^ mutation in cases with MAF by ddPCR (mean MAF = 3.7%), however, showed wild type with high MAF by ddPCR (18.2% and 19.36%). The ddPCR showed more comparable results to Sanger sequencing. PNA clamping assay had the possibility of lower specificity compare to ddPCR and Sanger sequencing.

In a comparison of Sanger sequencing and PNA-clamping assay, ddPCR with applied clinical cutoff eliminated false-positive results and preserved high sensitivity (100%) and specificity (100%) relative to the Sanger sequencing and PNA-clamping assay. In comparison with NGS panel sequencing (Supplementary Table S1), ddPCR and Sanger sequencing showed higher sensitivity (96.43% and 100%, respectively) and specificity (98.11% and 92.45%, respectively). NGS panel sequencing offered multiple gene screening including KRAS^G12/G13^ status, but showed a low sensitivity and positive predictive value. ddPCR, Sanger sequencing, and the PNA-clamping assay showed comparable results for detecting KRAS^G12/G13^ mutation, however, the required amount of DNA (1 μL) for ddPCR is much less than for Sanger sequencing (20 ng) and PNA-clamping assay (7 μL). This technical advantage is useful detecting KRAS^G12/G13^ mutation in small biopsied tissues or even liquid biopsy samples. Furthermore, the KRAS^G12/G13^ multiplex kit could not discriminate the mutation codon site and did not cover the full spectrum of mutation sites in KRAS. The ddPCR platform includes two fluorescence filters and supports at least duplex reactions. The development and optimization of higher-order multiplexing techniques for ddPCR are still required.

## 5. Conclusions

Determining KRAS mutational status has become crucial for successfully managing CRC patients, as well as in applications of anti-EGFR therapy. Furthermore, patients with the KRAS^G12/G13^ mutation tend to have more advanced tumors and shorter survival, implying that KRAS^G12/G13^ could be used as a prognostic factor [21]. Thus, KRAS^G12/G13^ is a highly informative and useful marker for the management of CRAC. By allowing the optimal cutoff value to be defined, ddPCR has potential for use as a diagnostic tool that could improve diagnostic sensitivity and specificity. Because ddPCR has high sensitivity and reproducibility, it would be suitable for daily application in laboratories seeking to detect KRAS^G12/G13^ mutations in CRAC tissue samples.

## Figures and Tables

**Table 1:** Diagnostic value of KRAS^G12/13^ mutation by ddPCR depending on mutant cutoff criteria.

	KRAS Mutant CRAC & Non-Neoplastic Colon (0.12% Cutoff)	KRAS Mutant and KRAS Wild CRAC & Non-Neoplastic Colon (7.9% Cutoff)
Sensitivity	100.0% (89.11–100.00)	84.38% (67.21–94.72)
Specificity	30.61% (18.25–45.42)	97.96% (89.15–99.95)
PPV	48.48% (43.87–53.13)	96.43% (79.42–99.47)
NPV	100.0% (100.00–100.00)	90.57% (81.09–97.23)

CRAC, colorectal adenocarcinoma; PPV, positive predictive value; NPV, negative predictive value.

**Table 2:** Repeated KRAS^G12/13^ mutation detection in CRAC tissue by ddPCR.

Sample ID	First KRAS ddPCR		Second KRAS ddPCR		Third KRAS ddPCR		Mean KRAS ddPCR	
	MAF		MAF		MAF		MAF	
T1	0	Wild-type	0	Wild-type	0	Wild-type	0	Wild-type
T2	0	Wild-type	0.07	Wild-type	0	Wild-type	0.02	Wild-type
T3	0.05	Wild-type	0	Wild-type	0	Wild-type	0.02	Wild-type
T4	0.05	Wild-type	0	Wild-type	0	Wild-type	0.02	Wild-type
T5	0.04	Wild-type	0	Wild-type	0.04	Wild-type	0.03	Wild-type
T6	0	Wild-type	0	Wild-type	0.11	Wild-type	0.04	Wild-type
T7	0.04	Wild-type	0.04	Wild-type	0.09	Wild-type	0.05	Wild-type
T8	0	Wild-type	0.19	Wild-type	0	Wild-type	0.06	Wild-type
T9	0.08	Wild-type	0.13	Wild-type	0	Wild-type	0.07	Wild-type
T10	0	Wild-type	0.14	Wild-type	0.1	Wild-type	0.08	Wild-type
T11	0.09	Wild-type	0.09	Wild-type	0.07	Wild-type	0.09	Wild-type
T12	0	Wild-type	0.15	Wild-type	0.11	Wild-type	0.09	Wild-type
T13	0.1	Wild-type	0.07	Wild-type	0.1	Wild-type	0.09	Wild-type
T14	0	Wild-type	0.13	Wild-type	0.19	Wild-type	0.11	Wild-type
T15	0	Wild-type	0.08	Wild-type	0.26	Wild-type	0.11	Wild-type
T16	0.11	Wild-type	0.27	Wild-type	0	Wild-type	0.12	Wild-type
T17	0	Wild-type	0.2	Wild-type	0.2	Wild-type	0.13	Wild-type
T18	0.02	Wild-type	0.15	Wild-type	0.27	Wild-type	0.15	Wild-type
T19	0.21	Wild-type	0.12	Wild-type	0.17	Wild-type	0.16	Wild-type
T20	0	Wild-type	0.13	Wild-type	0.36	Wild-type	0.16	Wild-type
T21	0.11	Wild-type	0.11	Wild-type	0.25	Wild-type	0.16	Wild-type
T22	0.09	Wild-type	0	Wild-type	0.4	Wild-type	0.16	Wild-type
T23	0	Wild-type	0.16	Wild-type	0.36	Wild-type	0.17	Wild-type
T24	0.07	Wild-type	0.11	Wild-type	0.33	Wild-type	0.17	Wild-type
T25	0	Wild-type	0.28	Wild-type	0.3	Wild-type	0.19	Wild-type
T26	0	Wild-type	0.46	Wild-type	0.12	Wild-type	0.19	Wild-type
T27	0.19	Wild-type	0.23	Wild-type	0.24	Wild-type	0.22	Wild-type
T28	0	Wild-type	0.48	Wild-type	0.26	Wild-type	0.25	Wild-type
T29	0.3	Wild-type	0.16	Wild-type	0.31	Wild-type	0.26	Wild-type
T30	0.28	Wild-type	0.19	Wild-type	0.41	Wild-type	0.29	Wild-type
T31	0.26	Wild-type	0.09	Wild-type	0.54	Wild-type	0.3	Wild-type
T32	0.06	Wild-type	0.53	Wild-type	0.3	Wild-type	0.3	Wild-type
T33	0	Wild-type	0.49	Wild-type	0.45	Wild-type	0.31	Wild-type
T34	0.34	Wild-type	0.23	Wild-type	0.39	Wild-type	0.32	Wild-type
T35	0.06	Wild-type	0.44	Wild-type	0.5	Wild-type	0.33	Wild-type
T36	0.07	Wild-type	0.12	Wild-type	0.79	Wild-type	0.33	Wild-type
T37	0.14	Wild-type	0.37	Wild-type	0.47	Wild-type	0.33	Wild-type
T38	0.04	Wild-type	0.55	Wild-type	0.58	Wild-type	0.39	Wild-type
T39	0	Wild-type	0.75	Wild-type	0.51	Wild-type	0.42	Wild-type
T40	0.27	Wild-type	0.4	Wild-type	0.76	Wild-type	0.48	Wild-type
T41	0	Wild-type	0.73	Wild-type	0.71	Wild-type	0.48	Wild-type
T42	0.22	Wild-type	0	Wild-type	1.4	Wild-type	0.54	Wild-type
T43	0.21	Wild-type	0.6	Wild-type	0.85	Wild-type	0.55	Wild-type
T44	0.7	Wild-type	1.08	Wild-type	0.69	Wild-type	0.82	Wild-type
T45	2.65	Wild-type	0	Wild-type	0	Wild-type	0.88	Wild-type
T46	0.97	Wild-type	0.8	Wild-type	0.97	Wild-type	0.91	Wild-type
T47	0.52	Wild-type	1.38	Wild-type	1.23	Wild-type	1.04	Wild-type
T48	3.69	Wild-type	1	Wild-type	0.66	Wild-type	1.78	Wild-type
T49	5.76	Wild-type	0.49	Wild-type	0.16	Wild-type	2.14	Wild-type
T50	5.79	Wild-type	1.04	Wild-type	0.42	Wild-type	2.42	Wild-type
T51	7.42	Wild-type	1.56	Wild-type	3.01	Wild-type	4	Wild-type
* T52	2.17	Wild-type	5.62	Wild-type	8.65	Mutant	5.48	Wild-type
* T53	3.61	Wild-type	9.11	Mutant	9.87	Mutant	7.53	Wild-type
* T54	5.48	Wild-type	7.74	Wild-type	10.49	Mutant	7.9	Mutant
T55	13.94	Mutant	15.08	Mutant	13.29	Mutant	14.1	Mutant
* T56	0.75	Wild-type	22.36	Mutant	22.88	Mutant	15.33	Mutant
T57	10.48	Mutant	17.56	Mutant	18.47	Mutant	15.51	Mutant
* T58	4.71	Wild-type	22.75	Mutant	21.34	Mutant	16.27	Mutant
T59	13.39	Mutant	20.33	Mutant	20.88	Mutant	18.2	Mutant
T60	16.62	Mutant	18.76	Mutant	21.23	Mutant	18.87	Mutant
T61	16.58	Mutant	17.12	Mutant	24.37	Mutant	19.36	Mutant
T62	15.04	Mutant	21.44	Mutant	22.65	Mutant	19.71	Mutant
T63	10.82	Mutant	24.39	Mutant	28.47	Mutant	21.23	Mutant
T64	9.23	Mutant	26.74	Mutant	28	Mutant	21.32	Mutant
* T65	2.04	Wild-type	29.01	Mutant	38.62	Mutant	23.22	Mutant
T66	15.63	Mutant	27.81	Mutant	29.46	Mutant	24.3	Mutant
T67	27.78	Mutant	23.06	Mutant	23.44	Mutant	24.76	Mutant
T68	24.88	Mutant	30.54	Mutant	31.24	Mutant	28.89	Mutant
T69	25	Mutant	32.61	Mutant	31.67	Mutant	29.76	Mutant
T70	9.47	Mutant	46.99	Mutant	50.34	Mutant	35.6	Mutant
T71	34.96	Mutant	34.48	Mutant	38.13	Mutant	35.85	Mutant
T72	20.1	Mutant	42.43	Mutant	45.5	Mutant	36.01	Mutant
T73	38.23	Mutant	32.7	Mutant	38.13	Mutant	36.36	Mutant
T74	36.08	Mutant	40.95	Mutant	43.71	Mutant	40.25	Mutant
* T75	4.29	Wild-type	55.56	Mutant	60.95	Mutant	40.27	Mutant
T76	30.18	Mutant	50.53	Mutant	49.34	Mutant	43.35	Mutant
T77	41.87	Mutant	42.24	Mutant	46.7	Mutant	43.6	Mutant
T78	41.14	Mutant	44.78	Mutant	48.95	Mutant	44.96	Mutant
T79	60.77	Mutant	45.28	Mutant	49.33	Mutant	51.79	Mutant
T80	53.88	Mutant	60.45	Mutant	58.23	Mutant	57.52	Mutant
T81	83.77	Mutant	78.03	Mutant	81.71	Mutant	81.17	Mutant

ddPCR, digital droplet PCR, * Cases yielded discrepant results regarding KRAS^G12/G13^ mutation status. MAF, mutant allele frequency.

**Table 3:** Diagnostic value of repeated measurement of KRAS^G12/13^ mutation by ddPCR.

	First ddPCR	Second ddPCR	Third ddPCR	Mean ddPCR
Sensitivity	71.88%	84.38%	84.38%	84.38%
Specificity	100%	97.96%	93.88%	97.96%
PPV	100%	96.43%	90.00%	96.43%
NPV	84.48%	90.57%	90.20%	90.57%

PPV, positive predictive value; NPV, negative predictive value.

**Table 4:** Comparative analysis of KRAS^G12/13^ mutation detection by ddPCR, Sanger sequencing, and peptide nucleic acid (PNA) clamping assay.

Sample ID	KRAS ddPCR	KRAS ddPCR	KRAS Sanger	KRAS PNA
	MAF	Cutoff Result	Sequencing	Clamping Assay
T1	0.00	Wild-type	Wild-type	Wild-type
T2	0.02	Wild-type	Wild-type	Wild-type
T3	0.02	Wild-type	Wild-type	Wild-type
T4	0.02	Wild-type	Wild-type	Wild-type
T5	0.03	Wild-type	Wild-type	Wild-type
T6	0.04	Wild-type	Wild-type	Wild-type
T7	0.05	Wild-type	Wild-type	Wild-type
T8	0.06	Wild-type	Wild-type	Wild-type
T9	0.07	Wild-type	Wild-type	Wild-type
T10	0.08	Wild-type	Wild-type	Wild-type
T11	0.09	Wild-type	Wild-type	Wild-type
T12	0.09	Wild-type	Wild-type	Wild-type
T13	0.09	Wild-type	Wild-type	Wild-type
T14	0.11	Wild-type	Wild-type	Wild-type
* T15	0.11	Wild-type	Wild-type	* Mutant
T16	0.12	Wild-type	Wild-type	Wild-type
T17	0.13	Wild-type	Wild-type	Wild-type
T18	0.15	Wild-type	Wild-type	Wild-type
T19	0.16	Wild-type	Wild-type	Wild-type
T20	0.16	Wild-type	Wild-type	Wild-type
T21	0.16	Wild-type	Wild-type	Wild-type
T22	0.16	Wild-type	Wild-type	Wild-type
* T23	0.17	Wild-type	* Mutant	Wild-type
T24	0.17	Wild-type	Wild-type	Wild-type
T25	0.19	Wild-type	Wild-type	Wild-type
T26	0.19	Wild-type	Wild-type	Wild-type
T27	0.22	Wild-type	Wild-type	Wild-type
T28	0.25	Wild-type	Wild-type	Wild-type
* T29	0.26	Wild-type	* Mutant	Wild-type
T30	0.29	Wild-type	Wild-type	Wild-type
T31	0.3	Wild-type	Wild-type	Wild-type
T32	0.3	Wild-type	Wild-type	Wild-type
T33	0.31	Wild-type	Wild-type	Wild-type
T34	0.32	Wild-type	Wild-type	Wild-type
T35	0.33	Wild-type	Wild-type	Wild-type
T36	0.33	Wild-type	Wild-type	Wild-type
T37	0.33	Wild-type	Wild-type	Wild-type
* T38	0.39	Wild-type	* Mutant	Wild-type
T39	0.42	Wild-type	Wild-type	Wild-type
T40	0.48	Wild-type	Wild-type	Wild-type
T41	0.48	Wild-type	Wild-type	Wild-type
* T42	0.54	Wild-type	* Mutant	Wild-type
T43	0.55	Wild-type	Wild-type	Wild-type
T44	0.82	Wild-type	Wild-type	Wild-type
T45	0.88	Wild-type	Wild-type	Wild-type
T46	0.91	Wild-type	Wild-type	Wild-type
* T47	1.04	Wild-type	Wild-type	* Mutant
* T48	1.78	Wild-type	* Mutant	Wild-type
* T49	2.14	Wild-type	Wild-type	* Mutant
* T50	2.42	Wild-type	Wild-type	* Mutant
* T51	4.00	Wild-type	Wild-type	* Mutant
* T52	5.48	Wild-type	Wild-type	* Mutant
* T53	7.53	Wild-type	Wild-type	* Mutant
* T54	7.90	Mutant	* Wild-type	Mutant
T55	14.1	Mutant	Mutant	Mutant
T56	15.33	Mutant	Mutant	Mutant
T57	15.51	Mutant	Mutant	Mutant
T58	16.27	Mutant	Mutant	Mutant
* T59	18.20	Mutant	Mutant	* Wild-type
T60	18.87	Mutant	Mutant	Mutant
* T61	19.36	Mutant	Mutant	* Wild-type
T62	19.71	Mutant	Mutant	Mutant
T63	21.23	Mutant	Mutant	Mutant
T64	21.32	Mutant	Mutant	Mutant
T65	23.22	Mutant	Mutant	Mutant
T66	24.30	Mutant	Mutant	Mutant
T67	24.76	Mutant	Mutant	Mutant
T68	28.89	Mutant	Mutant	Mutant
T69	29.76	Mutant	Mutant	Mutant
T70	35.60	Mutant	Mutant	Mutant
T71	35.85	Mutant	Mutant	Mutant
T72	36.01	Mutant	Mutant	Mutant
T73	36.36	Mutant	Mutant	Mutant
T74	40.25	Mutant	Mutant	Mutant
T75	40.27	Mutant	Mutant	Mutant
T76	43.35	Mutant	Mutant	Mutant
T77	43.60	Mutant	Mutant	Mutant
T78	44.96	Mutant	Mutant	Mutant
T79	51.79	Mutant	Mutant	Mutant
T80	57.52	Mutant	Mutant	Mutant
T81	81.17	Mutant	Mutant	Mutant

* Cases yielded discrepant results regarding KRAS^G12/G13^ mutation status. MAF, mutant allele frequency.

**Table 5:** Diagnostic value of KRA^G12/13^ mutation detection by ddPCR, Sanger sequencing, and PNA-clamping assay.

	Detection of KRA^G12/13^ Mutation		
	ddPCR	Sanger Sequencing	PNA-Clamping Assay
Sensitivity	100%	96.43%	92.86%
Specificity	100%	90.57%	86.79%
PPV	100%	84.38%	78.79%
NPV	100%	97.96%	95.83%

PPV, positive predictive value; NPV, negative predictive value.

## Supplementary Materials

The following are available online at https://www.mdpi.com/2077-0383/9/7/2283/s1: Table S1: Diagnostic value of KRA^G12/13^ mutation detection by ddPCR, Sanger sequencing, and PNA-clamping assay, and NGS panel sequencing (IonS5, Thermo Fisher Scientific).

## References

[1] Siegel R.L., Miller K.D., Jemal A. (2016). Cancer statistics, 2016. CA Cancer J. Clin..

[2] Siegel R.L., Miller K.D., Fedewa S.A., Ahnen D.J., Meester R.G., Barzi A., Jemal A. (2017). Colorectal cancer statistics, 2017. CA Cancer J. Clin..

[3] Malafosse R., Penna C., Cunha A.S., Nordlinger B. (2001). Surgical management of hepatic metastases from colorectal malignancies. Ann. Oncol..

[4] Alcaide M., Cheung M., Bushell K., Arthur S.E., Wong H.L., Karasinska J., Renouf D., Schaeffer D.F., McNamara S., Tertre M.C.D. (2019). A Novel Multiplex Droplet Digital PCR Assay to Identify and Quantify KRAS Mutations in Clinical Specimens. J. Mol. Diagn..

[5] Feng Q.-Y., Wei Y., Chen J.-W., Chang W.-J., Ye L.-C., Zhu D.-X., Xu J.-M. (2014). Anti-EGFR and anti-VEGF agents: Important targeted therapies of colorectal liver metastases. World J. Gastroenterol..

[6] Matsunaga M., Kaneta T., Miwa K., Ichikawa W., Fujita K.-I., Nagashima F., Furuse J., Kage M., Akagi Y., Sasaki Y. (2016). A comparison of four methods for detecting KRAS mutations in formalin-fixed specimens from metastatic colorectal cancer patients. Oncol. Lett..

[7] Rakhit C., Ottolini B., Jones C., Pringle J., Shaw J., Martins L.M. (2017). Peptide nucleic acid clamping to improve the sensitivity of Ion Torrent-based detection of an oncogenic mutation in KRAS. Matters.

[8] Lee H.S., Kim W.H., Kwak Y., Koh J., Bae J.M., Kim K.-M., Chang M.S., Han H.S., Kim J.M., Kim H.W. (2017). Molecular testing for gastrointestinal cancer. J. Pathol. Transl. Med..

[9] Miotke L., Lau B.T., Rumma R.T., Ji H.P. (2014). High sensitivity detection and quantitation of DNA copy number and single nucleotide variants with single color droplet digital PCR. Anal. Chem..

[10] Jones M., Williams J., Gartner K., Phillips R., Hurst J., Frater J. (2014). Low copy target detection by Droplet Digital PCR through application of a novel open access bioinformatic pipeline, ‘definetherain’. J. Virol. Methods.

[11] Trypsteen W., Vynck M., De Neve J., Bonczkowski P., Kiselinova M., Malatinkova E., Vervisch K., Thas O., Vandekerckhove L., De Spiegelaere W. (2015). ddpcRquant: Threshold determination for single channel droplet digital PCR experiments. Anal. Bioanal. Chem..

[12] Hughesman C.B., Lu X.J.D., Liu K.Y.P., Zhu Y., Poh C.F., Haynes C. (2016). A Robust Protocol for Using Multiplexed Droplet Digital PCR to Quantify Somatic Copy Number Alterations in Clinical Tissue Specimens. PLoS ONE.

[13] Dong L., Wang S., Fu B., Wang J. (2018). Evaluation of droplet digital PCR and next generation sequencing for characterizing DNA reference material for KRAS mutation detection. Sci. Rep..

[14] Vanova B., Kalman M., Jasek K., Kasubova I., Burjanivova T., Farkasova A., Kruzliak P., Busselberg D., Plank L., Lasabova Z. (2019). Droplet digital PCR revealed high concordance between primary tumors and lymph node metastases in multiplex screening of KRAS mutations in colorectal cancer. Clin. Exp. Med..

[15] Laurent-Puig P., Pekin D., Normand C., Kotsopoulos S.K., Nizard P., Perez-Toralla K., Rowell R., Olson J., Srinivasan P., Le Corre D. (2015). Clinical relevance of KRAS-mutated subclones detected with picodroplet digital PCR in advanced colorectal cancer treated with anti-EGFR therapy. Clin. Cancer Res..

[16] Yang W., Shelton D.N., Berman J.R., Zhang B., Cooper S., Tzonev S., Hefner E., Regan J.F. (2015). Droplet digital™ PCR: Multiplex detection of KRAS mutations in formalin-fixed, paraffin-embedded colorectal cancer samples. Biotechniques.

[17] Pinheiro L.B., Coleman V.A., Hindson C.M., Herrmann J., Hindson B.J., Bhat S., Emslie K.R. (2012). Evaluation of a droplet digital polymerase chain reaction format for DNA copy number quantification. Anal. Chem..

[18] Gerdes L., Iwobi A., Busch U., Pecoraro S. (2016). Optimization of digital droplet polymerase chain reaction for quantification of genetically modified organisms. Biomol. Detect. Quantif..

[19] Taylor S.C., Laperriere G., Germain H. (2017). Droplet Digital PCR versus qPCR for gene expression analysis with low abundant targets: From variable nonsense to publication quality data. Sci. Rep..

[20] Messa F., Tonissi F., Millo E., Bracco E., Ungari S., Lattanzio L., Merlano M., Damonte G., Lo Nigro C. (2014). A PNA-mediated clamping PCR for routine detection of KRAS mutations in colorectal carcinoma. Int. J. Biol. Markers.

[21] Dinu D., Dobre M., Panaitescu E., Bîrlă R., Iosif C., Hoara P., Caragui A., Boeriu M., Constantinoiu S., Ardeleanu C. (2014). Prognostic significance of KRAS gene mutations in colorectal cancer-preliminary study. J. Med. Life.

