# Supplementary material for: Identification of a Clinical Cutoff Value for Multiplex KRASG12/G13 Mutation Detection in Colorectal Adenocarcinoma Patients Using Digital Droplet PCR, and Comparison with Sanger Sequencing and PNA Clamping Assay
Source: J Clin Med. 2020 Jul 18;9(7):2283. doi: 10.3390/jcm9072283 (PMC7409004; doi:10.3390/jcm9072283)
Supplement: Supplementary file 1 [file jcm-09-02283-s001.pdf]

Supplementary Table S1. Diagnostic value of $KRA^{G12/13}$ mutation detection by ddPCR, Sanger sequencing, and PNA-clamping assay, and NGS panel sequencing (IonS5, Thermo Fisher Scientific).

	Detection of $KRA^{G12/13}$ Mutation			
	**ddPCR**	**Sanger sequencing**	PNA-clamping assay	NGS panel sequencing
Sensitivity	96.43%	100%	89.29%	39.29%
Specificity	98.11%	92.45%	84.91%	88.68%
PPV	96.43%	87.50%	75.76%	64.71%
NPV	98.11%	100%	93.75%	73.44%

PPV, positive predictive value; NPV, negative predictive value. * For comparative analysis, cases in which $KRAS^{G12/G13}$ mutation was detected by three or more methods were defined as positive references.
